# The Importance of H-FABP in Determining the Severity of Carbon Monoxide Poisoning

**DOI:** 10.4021/jocmr675w

**Published:** 2011-11-10

**Authors:** Ramazan Koylu, Basar Cander, Zerrin Defne Dundar, Oznur Koylu, Nazire Belgin Akilli, Korhan Ivelik

**Affiliations:** a Konya Training and Research Hospital, Emergency Department, Konya, Turkey; b Selcuk University Meram Faculty of Medicine, Emergency Medicine Department, Konya, Turkey; c Konya Training and Research Hospital, Biochemistry Department, Konya, Turkey

## Abstract

**Background:**

In this study, we aimed to investigate the importance of the use of heart-type fatty acid binding protein (H-FABP) in evaluating the myocardial damage in patients admitted to the emergency department with moderate to severe carbon monoxide (CO) poisoning.

**Methods:**

All patients admitted to the emergency department with severe acute CO intoxication were enrolled the study. The H-FABP and cardiac biomarker levels were assessed at 0, 6th and 24th hours. The patients were divided into groups as those with normal echocardiography findings and with wall motion abnormalities. The differences between the groups for these parameters were compared.

**Results:**

The mean age of 80 patients was 32.3 ± 12.9 years old. 42 of them were male. On admission, 29 (36.3%) had elevated serum troponin I levels and 56 (70.0%) had elevated serum H-FABP levels. At 6thhour, 4 (5.0%) of 80 patients had higher serum H-FABP levels and 23 (28.8%) of them had higher serum Troponin I levels than 0 hour. The patients with wall motion abnormality had significantly higher serum H-FABP levels compared to the patients with normal echocardiography findings at 6th and 24th hours (p = 0.001 and 0.009). While the serum COHb and H-FABP levels tended to decrease continuously in time (p < 0.001), the serum troponin I levels increased at 6th hour and then decreased at 24th hour (p = 0.017).

**Conclusion:**

The serum H-FABP levels are useful in identifying the myocardial damage in patients admitted to the emergency department with moderate to severe carbon monoxide poisoning at an early phase.

**Keywords:**

Carbon monoxide; Poisoning; H-FABP; Myocardial injury

## Introduction

Carbon monoxide (CO) is an odorless, colorless, tasteless, non-irritating gas formed as a by-product of burning organic compounds. The atmospheric concentration of CO is generally below 0.001%. The affinity of hemoglobin for CO is 200 to 250 times greater than its affinity for oxygen. This results in competitive inhibition of oxygen release due to a shift in the oxygen-hemoglobin dissociation curve, reduced oxygen delivery, and subsequent tissue hypoxia [1-3].

The body relies on oxygen to reach the cells to function; therefore, all the body systems are affected by carbon monoxide poisoning. The heart, the brain, and the lungs require continuously large amounts of oxygen for normal function and CO has its most profound impact on these vital organs, [2, 4-6]. Carbon monoxide poisoning has a variety of deleterious cardiac effects including arrhythmias, coronary spasm, ventricular dysfunction and myocardial infarction [7,8].

There is only limited correlation between the carboxyhemoglobin (COHb) level and the severity of clinical features [9]. Patients with pre-existing disease can experience increased exertional angina with COHb levels as low as 5-10%. At high COHb levels, even young healthy patients develop myocardial depression [10]. Recent studies suggest that a baseline electrocardiogram (ECG) should be performed and serial biomarkers should be followed-up in all patients with CO intoxication to determine the degree and duration of cardiac damage or dysfunction. Furthermore, patients with abnormal cardiac biomarkers should undergo an echocardiogram [7,11,12].

The heart-type fatty acid binding protein (H-FABP) is one of the most abundant proteins in the heart. H-FABP is a low molecular weight protein that behaves similarly to myoglobin in its kinetics and release. However, in contrast to myoglobin, there is more fatty acid binding proteins in the heart compared to skeletal muscle. It is more specific than myoglobin and it can be identified earlier than creatinin kinase MB (CK-MB) and troponins in acute coronary syndrome [13-16]. It has been reported in few clinical and experimental studies recently that H-FABP levels are useful in identifying the cardiotoxicity of carbon monoxide intoxications at an early phase [12,17].

In this study, we aimed to investigate the importance of the use of H-FABP in evaluating the myocardial damage in patients admitted to the emergency department with moderate to severe carbon monoxide poisoning.

## Methods

This prospective study was conducted, beginning from April 2008 to December 2010 in the intensive care unit of Konya Training and Research Hospital, Emergency Medicine Department. All patients (15 years of age and older) admitted to the emergency department with severe acute CO intoxication (COHb levels > 25%) were enrolled in the study.

The demographic characteristics of patients such as age and gender were recorded. Blood samples were collected on admission (hour 0) to the emergency department. For analysis of serum COHb, H-FABP, CK-MB and troponin I levels, 5 ml samples of venous blood drawn into vaccutainer tubes and heparinized syringes. 12-derivation electrocardiograms were performed on all patients on admission. Blood sampling and electrocardiograms were repeated at the 6th and the 24th hours for analysis of serum COHb, H-FABP, CK-MB, troponin I levels and electrocardiogram findings.

COHb levels were measured by a blood gas analyzer (Rapidlab® 1265) that was supported by a CO-oximetry panel developed by Siemens Healthcare Diagnostics. We used a sandwich enzyme-linked immunosorbent assay (ELISA) for the determination of human H-FABP (Cardiodetect®). The rapid H-FABP test was calibrated to detect a serum H-FABP concentration of > 7.0 ng/mL as a positive line. An automatic autoanalyzer was used in the biochemistry laboratory for determination of CK-MB and troponin I levels. Levels > 25 U/L for CK-MB and > 0.2 ng/mL for troponin I were accepted as indicating myocardial damage. Electrocardiograms were classified by rhythm and ST-T wave changes. Continuous ECG monitoring was performed on patients with chest pain, dyspnea, or ECG changes. Echocardiographies were performed and classified by wall motion abnormalities. Coronary angiography was performed on patients with ischemic ECG findings and elevated Troponin I levels at the 24th hour.

All the patients were conventionally treated using high-flow normobaric oxygen and monitored under intensive care conditions. All the patients were followed-up until the serum COHb levels decreased below 5% and their clinical findings such as headache, nausea, and vomiting disappeared.

Descriptive statistics were computed for age and gender. The data were expressed as mean ± SD. The differences in the means and the changes of the variables were tested using the Paired t test and the Student’s t test. The patients were divided into groups of those with normal echocardiography findings and those with wall motion abnormalities. For each group, the mean COHb, H-FABP, CK-MB, troponin I levels were computed for the 0 hour, 6th hour and the 24th hour. The differences between the groups for these parameters were compared using the Mann-Whitney U test. The relationship between COHb, H-FABP and troponin I levels was assessed using the Pearson correlation analysis. A p value of < 0.05 was accepted as statistically significant. The analysis of the data was performed using the SPSS version 16.0 (SPSS, Chicago, IL).

This study was conducted in accordance with the requirements of local ethical committee and was in compliance with the Helsinki Declaration.

## Results

A total of 80 patients were enrolled in the study during the study period. The mean age of patients was 32.3 ± 12.9 years. 42 (52.5%) of the 80 patients were male and 38 (47.5%) were female. The mean COHb levels were 39.7 ± 9.9% at hour 0, 8.2 ± 3.7% at the 6th hour and 1.5 ± 2.0% at the 24th hour. The COHb levels tended to decrease continuously at 0 hours, 6th hour and 24th hour, and this decrease was significant (p < 0.001).

On admission, 33 (41.2%) of the 80 patients had normal electrocardiograms. Sinus tachycardia was present in 39 (48.8%) of the 80 patients. 4 (5%) of them had ischemic changes, 3 (3.8%) had sinus arrhythmia, and 1 (1.2%) had ventricular extrasystole. At hour 6, 66 (82.5%) of the 80 patients had normal ECG and 75 (93.8%) of them had normal ECG at the 24th hour.

On admission, 23 (%28.8) of the 80 patients had elevated serum CK-MB levels (50.39 ± 42.64 U/L), 29 (36.3%) had elevated serum troponin I levels (0.71 ± 0.70 ng/mL), and 56 (70.0%) had elevated serum H-FABP levels (41.65 ± 20.35 ng/mL). At the 6th hour, 35 (43.8%) of the 80 patients had elevated serum CK-MB levels (47.17 ± 32.58 U/L), 33 (41.2%) had elevated serum troponin I levels (0.99 ± 1.26 ng/mL), and 35 (43.8%) had elevated serum H-FABP levels (30.34 ± 23.93 ng/mL). At the 24th hour, 27 (33.8%) of the 80 patients had elevated serum CK-MB levels (46.37 ± 26.39 U/L), 31 (38.8%) had elevated serum troponin I levels (0.78 ± 1.04 ng/mL), and 19 (23.8%) had elevated serum H-FABP levels (27.09 ± 29.32 ng/mL). At the 6th hour, 4 (5.0%) of the 80 patients had higher serum H-FABP levels than at 0 hour, and 23 (28.8%) of them had higher serum troponin I levels than at 0 hour.

Echocardiography was performed on 53 (66.3%) of the 80 patients. Those 53 patients were divided into 2 groups according to echocardiography findings. 41 (51.3%) of them had normal echocardiography findings (Group I) and 12 (15%) of them had wall motion abnormalities (Group II). There was no statistically significant difference between the groups in terms of serum COHb levels at 0 hour, 6th hour and the 24th hour (p > 0.05). There was no statistically significant difference between the groups in terms of serum H-FABP levels at 0 hour (p>0.05). However, the patients with wall motion abnormality had significantly higher serum H-FABP levels compared to the patients with normal echocardiography findings at the 6th hour and the 24th hour (respectively, p = 0.001 and 0.009) (Table 1).

**Table 1:** Comparison of the Patients With Normal Echocardiography Findings and With Wall Motion Abnormalities

	**Group I** **Normal echocardiography findings** **(n = 41) (mean ± SD)**	**Group II** **Wall motion abnormalities** **(n = 12) (mean ± SD)**	**p value**
COHb (%)			
Hour 0	39.57 ± 10.49	41.98 ± 12.79	p > 0.05
Hour 6	7.69 ± 3.26	7.83 ± 3.89	p > 0.05
Hour 24	1.24 ± 0.66	1.06 ± 0.65	p > 0.05
H-FABP (ng/mL)			
Hour 0	33.05 ± 23.73	41.17 ± 23.66	p > 0.05
Hour 6	12.37 ± 18.91	37.75 ± 31.99	p = 0.001
Hour 24	4.73 ± 11.56	24.75 ± 37.56	p = 0.009
Troponin I (ng/mL)			
Hour 0	0.25 ± 0.41	0.89 ± 0.93	p = 0.002
Hour 6	0.30 ± 0.43	1.69 ± 1.85	p = 0.003
Hour 24	0.25 ± 0.30	1.25 ± 1.57	p = 0.001
CK-MB (U/L)			
Hour 0	22.24 ± 13.99	57.58 ± 59.12	p = 0.002
Hour 6	26.24 ± 14.46	67.08 ± 47.49	p < 0.001
Hour 24	23.15 ± 14.79	51.08 ± 38.20	p = 0.002

**Figure 1:**
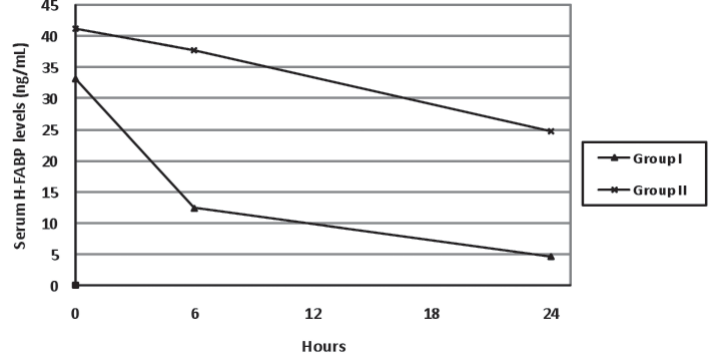
The changes in time in serum H-FABP levels.

The serum COHb and H-FABP levels tended to decrease continuously at 0 hour, 6th hour and 24th hour in both groups and this decrease was significant (group I, for both p < 0.001 and group II, respectively p < 0.001 and p = 0.012). The changes in time in serum H-FABP levels have been presented in Fig. 1.

**Figure 2:**
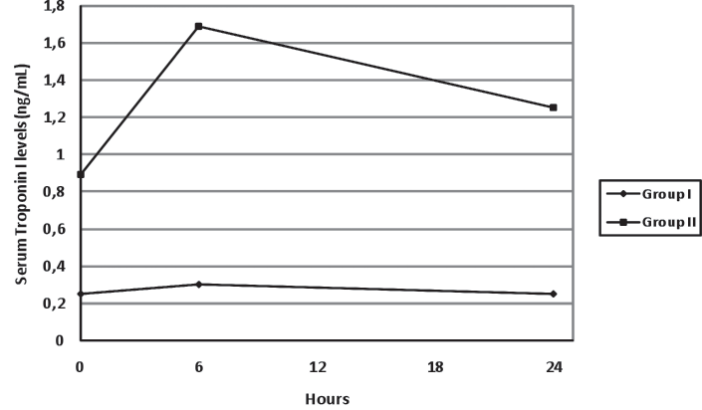
The change in time in serum troponin I levels.

The serum troponin I and CK-MB levels increased at the 6th hour and then decreased at the 24th hour in both groups, but the changes in serum troponin levels were statistically significant only in group II (p = 0.017). The change in time in serum troponin I levels has been presented in Fig. 2.

Only 3 (3.8%) patients underwent cardiac catheterization. None of them had specific coronary artery abnormalities on the coronary angiography.

The relationship between serum COHb levels at 0 hour and serum H-FABP, troponin I and CK-MB levels at 0 hour, 6th hour and 24th hour was assessed. No significant correlation was determined between serum COHb levels and CK-MB at 0 hour, 6th hour and 24th hour (p > 0.05). A weak significant correlation was found between the COHB levels and troponin I at 0 hour, (r = 0.386, p < 0.001) and H-FABP levels at 0 hour, 6th hour and 24th hour (for 0 hour, r = 0.227, p = 0.043; for the 6th hour, r = 0.312, p = 0.005; for the 24th hour, r = 0.407, p < 0.001).

77 (96.2%) of the 80 patients were discharged from hospital after the 24-hour follow-up period and 3 (3.8%) of them were discharged from hospital after 48 hours.

## Discussion

The presenting features of carbon monoxide poisoning are extremely vague. They include general malaise, headache, flu-like symptoms, gastroenteritis, lethargy, coma, convulsions, arrhythmias, and pulmonary edema, and generally there is only limited correlation between the COHb level and the severity of clinical features [9]. The prognosis of the patients with CO intoxication is due to the injury level of the two vital systems, nervous and cardiovascular systems. While several studies have been performed on the neurological impacts of CO poisoning and its effects on prognosis, recent studies have focused on cardiac injury [7,11,18,19]. Sustained myocardial injury due to CO poisoning has recently been demonstrated to be a significant predictor of mortality [10].

Myocardial injury due to CO poisoning results from tissue hypoxia, as well as damage at cellular level. Carbon monoxide may bind to proteins, including myoglobin and cytochrome-c-oxidase. The altered mitochondrial function secondary to hypoxia results in temporary contractile dysfunction of myocardial cells [7]. Other mechanisms of cell death secondary to carbon monoxide poisoning include oxygen free radical formation and subsequent lipid peroxidation along with induction of cellular apoptosis by nitric oxide secondary to high carbon monoxide levels [20]. In pre-clinical models, CO poisoning in dogs results in global as well as relative subendocardial hypoperfusion [21]. Cardiac injury at cellular level due to these probable causes appears as soon as contact with CO begins.

Monitoring markers including ECG, CK, CK-MB and troponin is recommended for the determination and follow-up of cardiac injury. Furthermore, echocardiography and coronary angiography are recommended for patients in whom signs of cardiac ischemia persist [7,18]. Recent studies support the use of the new biochemical indicators such as H-FABP and BNP in identifying the cardiotoxicity of CO intoxications at an early phase [12,18].

Human fatty acid-binding protein, a cytosolic protein abundantly present in myocardial tissue, plays a role in intracellular fatty acid transport [13]. Human fatty acid-binding protein is rapidly released into the circulation when the myocardium is injured. Its concentration starts to increase in 1.5 hours in the plasma, reaches a peak in 5-6 hours, and starts declining at the 24th hour. H-FABP is a new and valuable diagnostic instrument in identifying patients with myocardial injury, it is more specific for myocardium than myoglobulin, and like myoglobulin, its level increases in ischemia in the early phase [16,22,23].

There is only one experimental and one clinical study on H-FABP in CO poisoning. In a study with rats exposed to CO intoxication with two groups [mean COHB levels of 47% (group A) and 67% (group B)], it was reported that the serum H-FABP levels increased just after the CO exposure in both groups [17]. Furthermore, the H-FABP level was found to be higher in group B than in group A, immediately after the exposure. However, the serum troponin I levels only increased at 6 hours after the CO exposure in groups A and B. Similarly, we found that 70% of patients presenting to the Emergency Department (ED) with moderate or severe CO intoxication had elevated H-FABP levels, whereas 36% had elevated troponin I levels. H-FABP levels decreased at the 6th and the 24th hours while troponin I and CK-MB levels increased at the 6th hour and decreased at the 24th hour. These findings suggest that in CO intoxication, cardiac injury starts with exposure to CO and H-FABP can be used as an early marker of acute cardiac injury. However, in our study, the relationship between H-FABP and the duration of exposure could not be assessed, as there was lack of information on the duration and the severity of CO exposure. Further studies are necessary in this regard.

It was reported that ECG changes including atrial flutter, atrial fibrillation, tachycardia or ischemia can be found in CO intoxication, especially with carboxyhemoglobin levels of > 20-25% [6,7,18]. Various rates for ECG changes have been reported in patients with the diagnosis of CO intoxication. Acikalin et al. reported that 33% of their patients displayed ECG changes, whereas Satran et al. found in their study at a center for treatment of cases with moderate to severe CO poisoning using hyperbaric oxygen (HBO2) therapy that 84% of the patients had ECG changes [7,12]. Our findings showed that 41% of the patients had normal ECG findings on admission to the ED, and the most common pathological finding was sinus tachycardia with 48% of the patients. However, the patients with moderate to severe CO poisoning were enrolled in our study and further studies are required to determine the ECG change frequencies with every level of poisoning.

It was found in our study that 12 out of 53 patients (22.6%) with the diagnosis of CO poisoning had wall motion abnormality on the echocardiograms. While there was no significant difference between the two groups in terms of COHb levels, the h-FABP, troponin I and the CK-MB levels of these patients with wall motion abnormality on the echocardiograms were found to be significantly higher than that in patients with normal findings on the echocardiograms. The decrease in elevation of these cardiac biomarkers was slower in patients with wall motion abnormality. It was reported in several studies that CO intoxication causes transient wall motion abnormality and that the echocardiography should be part of the assessment for cardiac injury [7,11,19]. The lack of a significant difference in COHb levels in patients with and without pathology on the echocardiograms in our study suggests that there is no correlation between the COHb levels and cardiac injury. We can conclude that echocardiography may provide clinical information in the follow-up of patients with CO poisoning. However, this should be supported with further large-scale studies.

One of the most critical decisions in the management of patients presenting to the ED with CO intoxication is to decide which patients should be followed-up. While the previous follow-up algorithms created according to the COHb levels are under discussion, recent studies have reported that there is a poor relationship between COHb levels and the clinical presentation [9,10,19,24]. Our findings show that the H-FABP levels increase earlier than other cardiac biomarkers and that they are higher in patients with pathological results on echocardiography compared to patients with normal findings on echocardiography and show a slower and later decrease in patients with wall motion abnormalities. Again, when evaluated on a case basis, only four out of 80 patients (5%) in our study had higher H-FABP level at the 6th hour than that of at 0 hour; the troponin I levels of 23 patients (29%) were higher at the 6th hour than that of at 0 hour. The obtained data supports the use of H-FABP in identifying the cardiotoxicity in CO intoxications in patients presenting to the ED.

It has been reported that cardiac injury in CO intoxication is global and does not cause coronary artery pathology [7,11]. It was reported that high CK-MB and troponin I levels occurred in nearly 30% of patients with CO intoxication, although there were no obstructive lesions in the patients [11]. Despite the fact that approximately 40% of the 80 patients in our study had high levels of troponin I and CK-MB, three patients with persistent ECG changes and elevated enzyme levels underwent coronary angiography and no vascular pathology was found in these patients. This finding supports the probability of cardiac injury development in patients with normal coronary arteries following CO intoxication.

### Conclusion

Identification of the degree of cardiac injury is important in patients followed-up with the diagnosis of CO intoxication. ECG, cardiac enzymes, and echocardiography should be performed in the ED on these patients to determine the level of injury. Measurement of the level of H-FABP, a specific indicator in identifying the cardiac injury, is useful.

### Limitations

Our study has some limitations, including the lack of follow-up echocardiography, lack of information on the duration of exposure to CO, and the small number of patients with wall motion abnormality on echocardiography.
